# The Inflammatory Factor SNP May Serve as a Promising Biomarker for Acitretin to Alleviate Secondary Failure of Response to TNF-a Monoclonal Antibodies in Psoriasis

**DOI:** 10.3389/fphar.2022.937490

**Published:** 2022-06-24

**Authors:** Lanmei Lin, Yilun Wang, Xiaonian Lu, Tianxiao Wang, Qunyi Li, Runnan Wang, Jinfeng Wu, Jinhua Xu, Juan Du

**Affiliations:** ^1^ Department of Dermatology, Huashan Hospital Affiliated to Fudan University, Shanghai, China; ^2^ Department of Pharmacy, Huashan Hospital Affiliated to Fudan University, Shanghai, China

**Keywords:** psoriasis, biologics, single-nucleotide polymorphism (SNP), acitretin, secondary non-response

## Abstract

Psoriasis is a common immune-mediated inflammatory skin disease. Although biological agents have achieved good clinical efficacy in the treatment of moderate-to-severe psoriasis, the phenomenon of secondary non-response (SNR) has been gradually recognized. SNR refers to the gradual decline of efficacy after the patient achieves clinical remission with biological agents such as TNF-α biologics. Acitretin, as an immunomodulatory systemic drug for psoriasis, can improve the SNR to biological agents with good tolerance, but there are still individual differences in efficacy. Single-nucleotide polymorphisms (SNPs) of many related inflammatory cytokines have been shown to be important factors of individual differences in drug response in psoriasis, but there have been few reports on the use of pharmacogenomics to alleviate the SNR to biological agents. This study recruited 43 patients with psoriasis and 24 normal controls to investigate whether SNPs of inflammatory cytokines could be used as biomarkers for acitretin to alleviate SNR to TNF-α biologics in psoriasis, including rs1800795 (IL-6), rs6887695 (IL-12b), rs3212227 (IL-12b), rs10484879 (IL-17a), rs4819554 (IL-17ra), rs763780 (IL-17F), rs11209032 (IL23R), rs11209026 (IL23R), and rs2201841 (IL23R). The study also analyzed the correlation between the abovementioned SNPs and the efficacy of acitretin-only patients so as to understand whether the improvement is attributable to the intervention of acitretin on SNR or a simple response of acitretin. We found that in patients with homozygous AA (χ2 = 6.577, *p* = 0.02) at the SNP rs112009032 (IL-23R), acitretin could improve the SNR to TNFα monoclonal antibody. Patients with the genotype of TG (χ2 = 6.124, *p* = 0.035) at rs3212227 (IL-12B) were more sensitive to using acitretin in the treatment of psoriasis. Rs3212227 (χ2 = 7.664, *p* = 0.022) was also associated with the susceptibility to psoriasis. The study might provide a clinical decision reference for personalized treatment of secondary loss of response to psoriasis biologics.

## Introduction

Psoriasis is an autoimmune disease mediated by inflammatory cytokines. Genetic and environmental factors contribute to the disease together (Ray-Jones et al., 2016; Billi et al., 2019). Both innate immunity and adaptive immunity are involved in the pathogenesis, and inflammatory cytokines such as IL-17, IL-12, and IL23 run through the entire course (Girolomoni et al., 2012; Chiricozzi and Krueger, 2013). Biologics targeting inflammatory cytokines such as tumor necrosis factor (TNF)-α and interleukins (ILs) 12/23/17 have been used in the treatment of psoriasis (Mahil et al., 2016). While biologics are effective treatments, they are not always valid for all patients with psoriasis. During the long-term use of biological agents, the phenomenon of secondary non-response (SNR) to biological agents such as TNF-α monoclonal antibody (mab) has been gradually recognized (Rubbert-Roth et al., 2019). SNR refers to the gradual decline of efficacy after the patient achieves clinical remission with biological agents during the first six months. SNR is not uncommon even for the latest interleukin-17 monoclonal antibody (Weng et al., 2018). Part of the mechanism of SNR may be related to the production of the anti-drug antibodies, which is more common than primary unresponsiveness (Vallejo-Yagüe et al., 2021). It has been reported that the traditional systemic drugs (such as MTX and acitretin) can improve the secondary non-response to the biologics (Cather and Crowley, 2014). The combination therapy of MTX and biologics has been widely used to treat other inflammatory diseases, including rheumatoid arthritis (RA) and inflammatory bowel disease (IBD) (Busard et al., 2018). However, the adverse reactions of hepatotoxicity and gastrointestinal limit the clinical use of MTX (Cather and Crowley, 2014), and its combination with TNF-α mab increases the risk of reactivation of tuberculosis (Lorenzetti et al., 2014). Acitretin, as a traditional antiproliferative and immunomodulatory systemic drug for psoriasis, reduces the proliferation of keratinocytes, promotes the differentiation of keratinocytes, and also inhibits the induction of Th17 cells (Booij and Van De Kerkhof, 2011). It can also be used in patients with immunosuppression, such as those with infections or those susceptible to cancer (Gisondi et al., 2008). The combination of acitretin and biological agents can increase the efficacy of acitretin and also improve the SNR to biological agents (Gisondi et al., 2008) with good tolerance. But there are individual differences in the effect of acitretin (Cather and Crowley, 2014), especially in treating the SNR with the biologics, which should be investigated more precisely.

Single-nucleotide polymorphisms (SNPs) are the sequence polymorphisms of DNA caused by variation of a single nucleotide at the genome level, which is widespread in the human genome (Lander, 1996). Some SNPs have been used to identify genetic factors associated with complex diseases (Li et al., 2015), and more than 40 SNPs are confirmed to be related to psoriasis (Tsoi et al., 2012). The pro-inflammatory cells such as Th1 and Th17 cells are regulated at the genetic level (Farh et al., 2015). The SNPs of TNF-a (rs1799724 (Murdaca et al., 2017)), IL-6 (rs1800795 (Białecka et al., 2015)), IL-17 (rs10484879 (Murdaca et al., 2017), rs4819554 (Villalpando-Vargas et al., 2021)) IL-23 (rs11209032 (Karaderi et al., 2009), rs11209026 (Teng et al., 2015), rs2201841 (Zhu et al., 2012)), IL-12B (rs6887695 (Eiris et al., 2012), rs3212227 (Cargill et al., 2007)) and other pro-inflammatory cytokines are significantly associated with the pathogenesis of psoriasis. With the development of pharmacogenomics, genetic polymorphisms have been shown to be important factors of individual differences in drug response (Franke et al., 2008). Vinod Chandran et al.(Chandran et al., 2010) have shown that SNP (rs1232027) is related to the efficacy of methotrexate in the treatment of psoriatic arthritis, while the liver damage effect of cyclosporine (a medicine for the systemic treatment of psoriasis) has been shown to be related to CYP3A4*18B (Xin et al., 2014). Pharmacogenomics can be used to judge the efficacy of traditional systemic drugs or biological agents in the treatment of psoriasis (Murdaca et al., 2017; Ovejero-Benito et al., 2018), but there have been few reports on the use of pharmacogenomics to alleviate the SNR to biological agents. In order to screen out patients who are more suitable for using acitretin to reduce SNR to TNF-a monoclonal antibody, the study analyzed the relationship between different inflammatory cytokine-related SNPs and the therapeutic effect of acitretin alleviating SNR, so as to identify relevant genetic prognostic biomarkers and provide a reference for individualized treatment of patients.

## Materials and Methods

### Subjects

**FIGURE 1:**
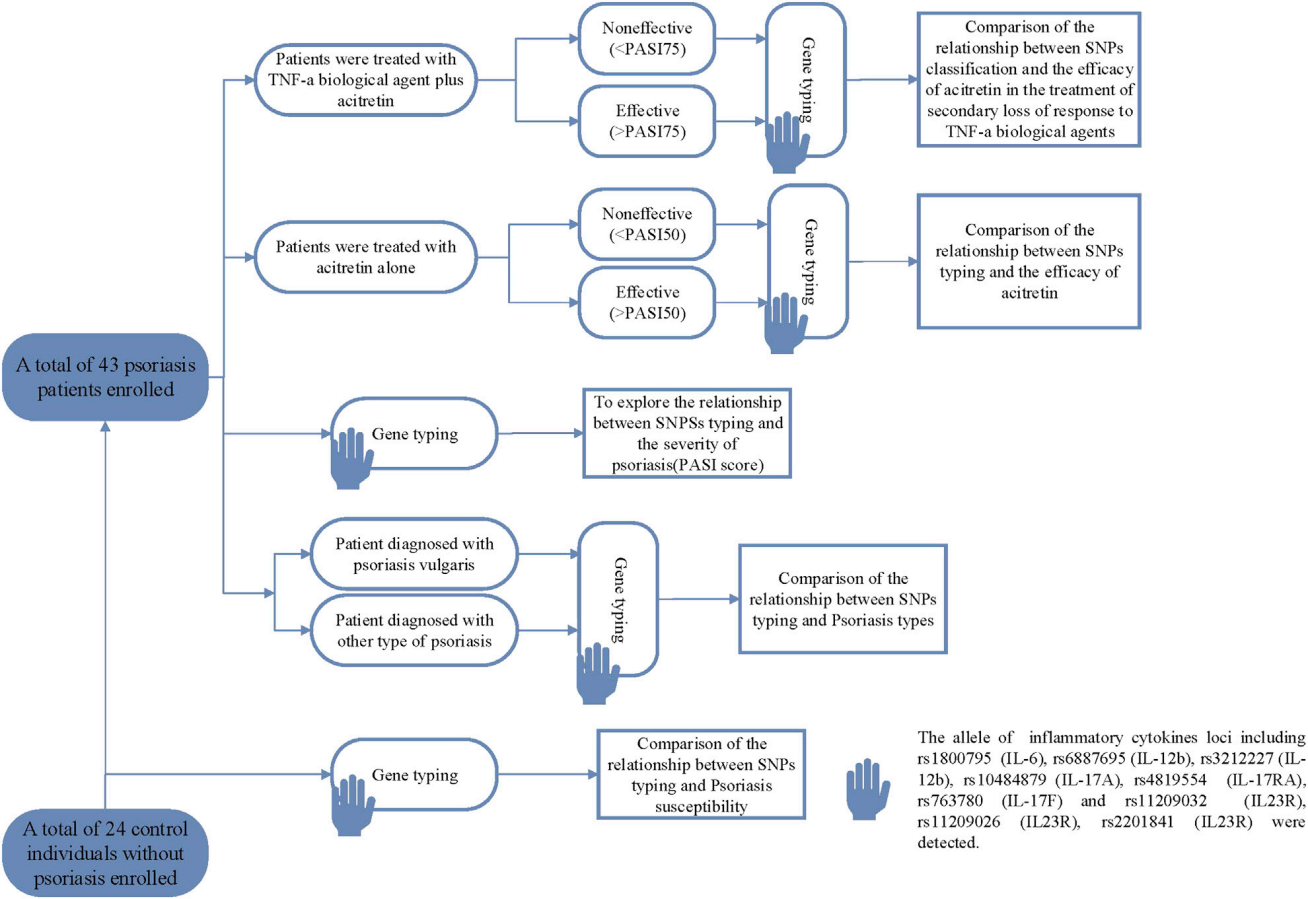
The flowchart of clinical trials and analysis in this study.

The study was retrospective, including 43 patients with psoriasis and 24 normal controls. As shown in Figure 1, the patients with psoriasis were divided into two groups: patients who used acitretin after the secondary loss of response to TNF-α mab, and patients who used acitretin-only. In order to find out the relationship between different SNPs and the effect of acitretin alleviating SNR on biological agents, the study detected and analyzed the differences in SNPs between the effective and ineffective patients within each treatment group. Patients with SNR to TNF-α mab were retrospectively assessed by experienced dermatologists. SNR referred to the gradual decline of efficacy after the patient achieves clinical remission with TNF-α mab during the first 6 months. Recruited psoriasis patients were based on the following inclusion criteria: 18 years of age or older and their clinically diagnosed course of psoriasis was at least 6 months. TNF-α mab we used in the study included 16 cases of adalimumab and 3 cases of etanercept. The Exclusion criteria of patients were as follows: drug-induced psoriasis; the presence of skin diseases other than psoriasis that might interfere with clinical assessment (eg, eczema); somatic diseases which significantly reduced the immunity of patients (hematological diseases, neurological diseases, infectious diseases, liver diseases, kidney diseases, and lung diseases). The PASI score (Oakley, 2009) was used to evaluate the clinical severity of psoriasis. According to the different treatment goals of traditional systemic drugs and the biologics of psoriasis in the guidelines and literature, PASI50 was defined as effective in patients who only used acitretin for treatment, and PASI75 was defined as effective in patients who used acitretin after secondary non-response to TNF-α mab (Chiu and Tsai, 2019).

### Gene Typing

5 ml of venous blood was drawn from each participant for genotyping. All blood samples were stored at −80°C until use. DNA was extracted using the Ezup Column Blood Genomic DNA Extraction Kit according to the manufacturer’s protocol (Sangon biotech, B518253). The allele of psoriasis-susceptible inflammatory cytokines loci including rs1800795 (Białecka et al., 2015) (IL-6), rs6887695 (Eiris et al., 2012) (IL-12b), rs3212227 (Cargill et al., 2007) (IL-12b), rs10484879 (Murdaca et al., 2017) (IL-17A), rs4819554 (Villalpando-Vargas et al., 2021) (IL-17RA), rs763780 (Villalpando-Vargas et al., 2021) (IL-17F) and rs11209032 (Karaderi et al., 2009) (IL23R), rs11209026 (Teng et al., 2015) (IL23R), rs2201841 (Zhu et al., 2012) (IL23R) were detected. SNPs were genotyped by allele-specific matrix-assisted laser desorption/ionization time-of-flight mass spectrometry (MassARRAY^®^ MALDI-TOF System).

### Statistical Analysis

All analyses were performed using SPSS 18.0 statistical software package (IBM SPSS, IL, United States). The frequency distribution of alleles in different subgroups was tested by Fisher’s exact test analysis. Analysis of variance was used to analyze different allele and genotype frequencies and PASI baseline scores. Efficacy comparisons between different genotypes and patients were analyzed using the Kruskal–Wallis test. Two-tailed *p*-values less than 0.05 were considered significant.

## Results

### Epidemiological Data of the Participants and the Association Between Their Detected SNPs and Psoriasis

A total of 43 patients with psoriasis (19 patients treated with TNF-α mab and acitretin, 24 patients treated with acitretin only) and 24 normal controls were included in this study. The enrolled patients with psoriasis were all severe (PASI score 25.45 ± 12.66), of which 35 were psoriasis vulgaris, three were erythrodermic psoriasis, two were arthritic psoriasis, two were pustular psoriasis, and one was psoriasis guttate. The average age of the psoriasis patient was 52.56 ± 16.13, including 25 males and 18 females. The average age of the normal controls was 58.83 + 10.44, including 11 males and 13 females. There was no statistically significant difference in the distribution of age and gender between patients and normal controls (Table 1). The enrolled individuals were genotyped for the SNPs including rs1800795 (IL-6), rs6887695 (IL-12b), rs3212227 (IL-12b), rs10484879 (IL-17A), rs4819554 (IL-17RA), rs763780 (IL-17F), rs11209032 (IL23R), rs11209026 (IL23R), and rs2201841 (IL23R). There was no significant difference between SNPs rs11209026, rs10484879, and rs1800795 between the two groups. It was suggested that the population of GG genotype at the IL-12b rs3212227 variants was dominated by normal controls rather than the psoriasis population (χ2 = 7.664, *p* = 0.022), and the T allele showed a higher frequency in the psoriasis population (*p* = 0.036). The allele and genotype frequencies of the other five SNPs (rs6887695, rs4819554, rs763780, rs11209032, and rs2201841) were not statistically associated with psoriasis susceptibility (Table 2). None of the selected SNPs was found to be associated with the severity and type of psoriasis (Table 3).

**TABLE 1:** The demographics data of psoriasis patients and controls.

	N	Male/female	Age (mean ± SD)	PASI
**Psoriasis**	43	25/18	52.56 ± 16.13	25.45 ± 12.66
**Control**	24	11/13	58.83 + 10.44	—
*p* **value**	—	0.333	0.058	—
—	**Adalimumab**	**Etanercept**	**Acitretin**
**N (%)**	16 (37.20%)	3 (6.66%)	24 (55.81%)

**TABLE 2:** Association of the alleles at SNPs and the genotype frequency with susceptibility to psoriasis.

SNP	Genotype/allele	Psoriasis	Controls	Value	Sig	OR (95%CI)
Rs763780	TT	34	20	1.785	0.410	—
	CT	6	4	—	—	—
	CC	3	0	—	—	—
	T	74	44	0.925	0.336	0.561 (0.170–1.846)
	C	12	4	—	—	1.00 (References)
Rs4819554	AG	13	12	3.899	0.142	
	AA	21	6	—	—	—
	GG	9	6	—	—	—
	A	55	24	2.479	0.115	1.774 (0.866–3.634)
	G	31	24	—	—	—
Rs11209032	AA	11	6	0.026	0.987	—
	AG	22	12	—	—	—
	GG	10	6	—	—	—
	A	44	24	0.017	0.897	1.048 (0.517–2.123)
	G	42	24	—	—	—
Rs6887695	CC	4	5	3.358	0.187	—
	CG	22	14	—	—	—
	GG	17	5	—	—	—
	C	30	24	2.926	0.087	0.536 (0.261–1.099)
	G	56	24	—	—	—
Rs3212227	GG	3(a)	8 (b)	7.664	0.022*	—
	TT	12 (a)	4 (a)	—	—	—
	TG	28(a)	12 (a)	—	—	—
	G	34	28	4.379	0.036*	0.467 (0.228–0.958)
	T	52	20	—	—	—
Rs2201841	GG	29	15	2.681	0.303	—
	AG	13	6	—	—	—
	AA	1	3	—	—	—
	A	71	36	1.094	0.296	1.578 (0.669–3.723)
	G	15	12	—	—	—
Rs1800795	GG	41	24	—	1.000	—
	CG	2	0	—	—	—
	G	84	48	—	1.000	0.636 (0.559–0.724)
	C	2	0	—	—	—
Rs11209026	GG	43	24	—	—	—
RS10484879	GG	43	24	—	—	—

(a), (b), (a, b): No significant differences existed between any groups with the same letters, and significant differences existed only if the letters were completely different.

**TABLE 3:** The relationship between each SNP and the inflammatory degree (PASI score)/different types of psoriasis.

SNP	Genotype	N	Mean (PASI)	K-W H	Sig	Psoriasis vulgaris	Other type of psoriasis	χ2	Sig
Rs763780	TT	34	24.46	2.027	0.363	27	7	0.789	0.674
	CT	6	27.30	—	—	5	1	—	—
	CC	3	32.50	—	—	3	0	—	—
Rs4819554	AG	13	21.87	2.160	0.340	11	2	0.364	1.000
	AA	21	28.54	—	—	17	4	—	—
	GG	9	23.41	—	—	7	2	—	—
Rs11209032	AA	11	27.08	1.072	0.585	7	4	2.771	0.330
	AG	22	26.00	—	—	19	3	—	—
	GG	10	22.44	—	—	9	1	—	—
Rs6887695	CC	4	25.08	0.509	0.775	3	1	1.180	0.518
	CG	22	24.23	—	—	17	5	—	—
	GG	17	27.11	—	—	15	2	—	—
Rs3212227	GG	3	24.33	2.478	0.290	2	1	0.962	0.679
	TT	12	30.63	—	—	10	2	—	—
	TG	28	23.35	—	—	23	5	—	—
Rs2201841	GG	29	26.14	2.948	0.229	24	5	0.831	0.744
	AG	13	23.23	—	—	10	3	—	—
	AA	1	34.2	—	—	1	0	—	—

### Relationship Between SNP Genotype and the Effect of Acitretin Reducing SNR to TNF-α Mab

The patients who used acitretin after SNR to TNF-α mab (T&A) were divided into an effective group and a noneffective group. There were four patients in the effective group, and their PASI score improved 84.90 ± 10.48% compared with the baseline. There were 15 patients in the non-effective group, and the PASI score improved by 56.39 ± 9.37%. The patients who were treated with acitretin only (A) were also divided into an effective group and a noneffective group. There were six patients in the effective group, and the PASI score improved 59.89 ± 5.10% compared with the baseline. There were 18 patients in the non-effective group, and the PASI score improved by 33.95 ± 11.88%. There was difference at IL-23R SNP (rs11209032, χ2 = 6.577, *p* = 0.02) between the effective group (T&A) and the non-effective group (T&A). The frequency of AA genotype in the effective group (T&A) was higher than that in the noneffective group (66.7%). No correlation between rs11209032 and the efficacy was found in patients who used acitretin only, so the effect of this SNP on the sensitivity of acitretin was excluded. Moreover, in patients treated with acitretin alone (A), the SNP (rs3212227, χ2 = 6.124, *p* = 0.035) at the IL-12b was significantly different between the effective group and the non-effective group. The frequency of the TG (50.0%) genotype was significantly higher than that of the GG (16.7%) genotype in the effective group (A). The frequency of the GG (72.20%) genotype in the non-effective group (A) was significantly higher than that of the TG (11.1%) genotype. The other SNPs were not found to be associated with the efficacy of acitretin alleviating SNR or acitretin alone (Table 4).

**TABLE 4:** Comparison of the relationship between each SNP and the efficacy of acitretin alone or the efficacy of acitretin treating secondary non-response to TNF-a biological agents.

SNP	Therapy	Genotype	Noneffective	Effective	χ2	Sig
Rs763780	T&A	TT	12	1	5.096	0.071
		CT	1	2	—	—
		CC	2	1	—	—
	A	TT	17	4	—	0.143
		CT	1	2	—	—
Rs4819554	T&A	AG	5	2	2.348	0.480
		AA	4	2	—	—
		GG	6	0	—	—
	A	AG	4	3	2.150	0.391
		AA	10	3	—	—
		GG	4	0	—	—
Rs11209032	T&A	AA	1 (a)	2 (a)	6.577	0.020*
		AG	10 (b)	0 (b)	—	—
		GG	4 (a, b)	2 (a, b)	—	—
	A	AA	5	3	1.317	0.699
		AG	10	2	—	—
		GG	3	1	—	—
Rs6887695	T&A	CC	2	0	1.393	0.740
		CG	7	1	—	—
		GG	6	3	—	—
	A	CC	1	1	1.214	0.792
		CG	11	3	—	—
		GG	6	2	—	—
Rs3212227	T&A	TT	5	2	—	0.603
		TG	10	2	—	—
	A	GG	13 (b)	1 (a)	6.124	0.035*
		TT	3 (a)	2 (a)	—	—
		TG	2 (b)	3 (a)	—	—
Rs2201841	T&A	GG	8	2	0.723	1.000
		AG	6	2	—	—
		AA	1	0	—	—
	A	GG	14	5	—	1.000
		AG	4	1	—	—

T&A: treated with TNF-a, monoclonal antibody plus acitretin. A: treated with acitretin alone. (a), (b), (a, b): no significant differences existed between any groups with the same letters, and significant differences existed only if the letters were completely different.

## Discussion

With the continuous development of molecular biology, the research on inflammatory pathways in the pathogenesis of psoriasis has gradually become precise. The biological therapies targeting the inflammatory cytokines such as tumor necrosis factor (TNF)-α and interleukins (ILs)-12/23/17 have shown good efficacy, which have been the first-line systemic treatment for psoriasis in many national guidelines (Mahil et al., 2016; Amatore et al., 2019; Smith et al., 2020; Nast et al., 2021). Although their effect on most patients is good, there are still some patients with limited response to the biologics, which is called primary nonresponse. Secondary non-response refers to the gradual decline of efficacy after the patient achieves clinical remission with biological agents during the first 6 months (Vallejo-Yagüe et al., 2021). For patients with SNR to the biologics, current interventions include increasing the dose or switching to another therapy, such as adding traditional systemic drugs. Methotrexate is an immunosuppressant in combination with a TNF-α inhibitor that can eliminate antidrug antibodies and restore clinical response (Ben-Horin et al., 2013), but hepatotoxicity and reactivation of tuberculosis of methotrexate greatly limit its use (Lorenzetti et al., 2014; Vallejo-Yagüe et al., 2021). Therefore, our study turned attention to acitretin. As a routine clinical drug for psoriasis, the effectiveness of acitretin in alleviating SNR to the biological agents has been reported (Gisondi et al., 2008; Cather and Crowley, 2014). In patients with refractory psoriasis, the combination of acitretin and the TNF-α monoclonal antibody like etanercept has achieved a good result (Armstrong et al., 2015). In the era of biologics treating psoriasis, it is required more precise guidance in order to reduce the possible drug resistance and economic losses caused by random drug switches. An increasing number of studies are dedicated to finding predictors to guide the dosing of biologics (Gisbert and Chaparro, 2020), and SNP is one of the common biomarkers (Franke et al., 2008). To take advantage of the robust SNP data and to better understand the genetic susceptibility to psoriasis, our study utilized known risk genes to search for novel prognostic markers for acitretin reducing secondary non-response to TNF-α mab.

This study included the patients who received acitretin after the secondary non-response to the TNF-a inhibitor. Since the relationship between the efficacy of TNF-α monoclonal antibodies and different inflammatory SNPs has been reported, it could be used to further exclude the potential impact of different SNPs on the sensitivity of TNF-α mab (van den Reek et al., 2017; Murdaca et al., 2017). In order to distinguish that the improvement in efficacy was due to the acitretin alleviating SNR to TNF-α mab rather than the response of acitretin itself, we also included patients who received acitretin-only for comparison. In the traditional systemic therapy, patients were defined as remission when they improved more than 50% compared to the baseline PASI score (PASI50). With the clinical use of biological agents, the overall efficacy of psoriasis has been greatly improved, and the treatment goals have also increased. PASI75 has been used as an indicator to evaluate the therapeutic effect of psoriasis biological agents (Chiu and Tsai, 2019). Therefore, we used different efficacy-judging indicators in the acitretin alleviating SNR in the TNF-α mab group and the acitretin-only group. For patients with SNR to TNF-α mab, we found that the genotype of rs11209032 (IL-23R) was biased towards an AA in patients who responded to the combination therapy, and the genotype of rs11209032 was biased towards AG in patients who did not respond to the therapy. But we did not find a meaningful association of rs11209032 in acitretin-only patients. Therefore, we suggested that in patients with AA genotype of rs11209032, acitretin can better alleviate the SNR to TNF-α monoclonal antibody, which was not due to the effect of acitretin itself. The correlation between the efficacy of TNF-α monoclonal antibody and IL-23R genetic polymorphism has not been found in the current pharmacogenomics studies, so the potential impact of rs11209032 on the initial response of biologics could be excluded (van den Reek et al., 2017; Murdaca et al., 2017). In patients treated with acitretin alone, the genotype of rs3212227 (IL-12B) was biased to genotype TG in patients who had achieved PASI50 and genotype GG in patients who did not achieve PASI50. While rs3212227 was also significantly different in the distribution of psoriasis and normal controls (χ2 = 7.664, *p* = 0.022), and T allele also showed a higher frequency in the psoriasis population (*p* = 0.036) (χ2 = 6.317, *p* = 0.048). IL-12 and IL-23 play important roles in the pathogenesis of psoriasis by sharing the p40 subunit required for binding to their receptors which have been shown to be overexpressed in psoriatic lesions. IL-12 and IL-23 can induce cells to differentiate into Th1/Th17, thereby increasing the production of pro-inflammatory cytokines, including IL-17A, IL-17F, IL-22, IL-26, IFN-γ, CCL20, and TNF-α(Jeon et al., 2017). In addition, the role of interleukin 12/23p40 cytokines in psoriasis and other inflammatory diseases is also supported by the effectiveness of interleukin 12/23 mAb therapy (Eiris et al., 2012). Rs11209032 has been shown to be associated with many diseases, including psoriasis, Behcet’s disease, and ankylosing spondylitis and is related to the efficacy of immunotherapy in aplastic anemia (Jiang et al., 2010; Roberts et al., 2016; Zhao et al., 2018). Rs3212227 has been extensively studied in diseases (for example, rheumatoid arthritis (Shen et al., 2015), cervical cancer (Chen et al., 2009)) and the possibility of being a relevant marker of prognosis has been proposed in different tumors (Cerhan et al., 2007; Yuzhalin and Kutikhin, 2012). Therefore, this study suggested that rs11209032(IL-23R) and rs3212227(IL-12B) may be used as genetic biomarkers for clinic use. In patients with psoriasis, the genotype of rs11209032 can be detected to determine whether acitretin is suitable to reduce the SNR and the genotype of rs3212227 can also be detected to know their response to acitretin in advance. In addition, there was no significance of rs11209026, rs10484879, rs1800795, rs6887695, rs4819554, rs763780, and rs2201841 between each group. The sample size may need to be further expanded.

The combination of traditional medications and biologics has potential synergistic effects. In addition to alleviating SNR to the biological agents, combination therapy can improve drug efficacy, accelerate the remission and reduce the costs of disease (Cather and Crowley, 2014). Improving the long-term efficacy of TNF-α monoclonal antibodies can reduce the incidence of cardiovascular events and mortality (Hugh et al., 2014; Rubbert-Roth et al., 2019). In addition, since the elevation of psoriasis-related inflammatory cytokines may also be associated with other inflammatory comorbidities (Cather and Crowley, 2014), enhancing the long-term efficacy of monoclonal antibodies may also provide broader benefits for patients.

## Conclusion

Our study investigated the possibility of inflammatory cytokines SNPs as biomarkers for the prediction of acitretin alleviating SNR to TNF-α mab. We found that in patients with homozygous AA at the SNP rs112009032 (IL-23R), acitretin could improve the SNR to TNF-α monoclonal antibody. Patients with the genotype of TG at rs3212227 (IL-12B) were more sensitive to using acitretin in the treatment of psoriasis. Rs3212227 was also associated with the susceptibility to psoriasis. The study is an exploration of using the data of SNPs to provide a clinical decision reference for personalized treatment of secondary loss of response to psoriasis biologics.

## Data Availability

The original contributions presented in the study are included in the article/Supplementary Material, further inquiries can be directed to the corresponding authors.
